# Quercetin promotes production of secondary hair follicle stem cells in cashmere goat: a mechanistic study

**DOI:** 10.3389/fvets.2025.1689059

**Published:** 2025-10-31

**Authors:** Wei Lian, Guoqing Jiang, Xueyong Wu, Yadong Gao, Kun Cui, Lei Zhu, Ziyang Xu, Xiao Zhang, Jiawei Wang, Mingli Peng, Rui Ding, Fei Hao, Dongjun Liu

**Affiliations:** Key Laboratory of Reproductive Regulation and Breeding of Grassland Livestock, School of Life Sciences, Inner Mongolia University, Hohhot, China

**Keywords:** quercetin, hair follicle stem cell, cell proliferation, apoptosis, antioxidation, RNA-Seq, cashmere goat

## Abstract

**Introduction:**

This study investigated the effects of quercetin on the proliferation and apoptosis of secondary hair follicle stem cells (SHFSCs) isolated from Arbas cashmere goats.

**Methods:**

SHFSCs were treated with varying quercetin concentrations. CCK-8, EdU assays, and flow cytometry analyses were performed to assess cell proliferation and apoptosis. Transcriptome analyses were used to identify differentially expressed genes and enriched signaling pathways.

**Results:**

Treatment with 10 μg/mL quercetin for 48 h significantly promoted cell proliferation. The proportion of S-phase cells increased from 15.5% to 21.2%, and the mRNA and protein levels of PCNA and TERT were upregulated. Quercetin inhibited apoptosis by downregulating BAX, TP53, and CASP3, upregulating BCL2, and reducing the number of late apoptotic cells. Mechanistically, quercetin activated the PI3K–Akt, Wnt, and TGF-β signaling pathways, upregulated CCND1 and CDK4 expression, improved mitochondrial membrane potential, reduced ROS levels, and promoted VEGF, FGF, and HGF secretion. Transcriptome analyses revealed that differentially expressed genes were enriched in translational processes, insulin-like growth factor binding, and proliferation-related signaling pathways.

**Conclusion:**

Quercetin promotes SHFSC proliferation and inhibits apoptosis through multiple pathways, providing a potential regulatory strategy for improving cashmere production in goats.

## 1 Introduction

Arbas cashmere, a key raw material for down products, is renowned for its warmth, lightness, and bright luster (1). The Arbas cashmere goat, a unique breed from Inner Mongolia, has long been globally recognized for its advantages in terms of cashmere yield, fineness, and length (2). The goat's hair follicles, an appendage of the skin, play a crucial role in determining cashmere quality. Cashmere goat hair follicles are categorized into primary (hair-producing) and secondary follicles (cashmere-producing) (3). Research on secondary follicles is essential for improving the quality of cashmere. The seasonal cycle of secondary hair follicles in adult cashmere goats typically spans anagen (April–November), catagen (December–January), and telogen (February–March) phases (4, 5). By contrast, during the fetal period and early postnatal stage, secondary follicles primarily undergo morphogenesis and maturation. The present study investigates adult animals Anagen phase directly influences cashmere yield. Previous studies have shown that secondary hair follicle stem cells (SHFSCs), located in the bulge region adjacent to the arrector pili muscle at the base of the follicles, are critical for follicle growth and development. SHFSCs maintain their stemness and regulate follicle growth cycles through interactions with the surrounding microenvironment, including the dermal papilla (DP) cells, the extracellular matrix, and various signaling molecules (4, 6, 7).

As cashmere is produced only by secondary follicles, SHFSCs are the key cellular drivers of cashmere output. Their proliferation–apoptosis balance controls follicular phase transitions and directly shapes fiber yield, fineness, and length. Thus, SHFSC-focused studies can provide a more precise mechanistic view than whole-follicle approaches for developing tractable cellular targets to boost production.

At cellular level, SHFSCs play a key role in follicular development. During the anagen phase, DP cells secrete key signals, including Wnt ligands. Activation of Wnt signaling pathway stimulates SHFSC activity, promotes proliferation, and sustains stemness. For example, WNT10b induces anagen (8, 9), driving SHFSCs proliferation, differentiation, and initiating follicle cycle progression (10). During catagen phase, bone morphogenetic protein (BMP) signaling inhibits SHFSCs activation, regulating the cells into a quiescent state to prevent premature differentiation and maintain the stem cell pool, with BMP4 and BMP6 playing important roles (9, 11, 12). The JAK/STAT pathway modulates stem cell behavior under specific conditions, while the PI3K/AKT pathway promotes SHFSCs growth and differentiation and inhibits apoptosis, balancing quiescence and activation (13–15). TGF-β signaling participates in regulating inflammation and cellular responses during follicle regeneration (16–18). Additionally, the transcription factor RUNX1 enhances SHFSC sensitivity to activation signals, promoting the transition from telogen to anagen, while the vascular system interacts with hair stem cells to maintain follicle cycle homeostasis (19–21). Despite systematic studies on the regulatory mechanisms underlying SHFSC behavior, the complex follicle regulatory network requires further exploration to develop effective modulation strategies.

Quercetin, a secondary metabolite abundant in onions, grapes, and tea, exhibits antioxidant, anti-inflammatory, and anticancer properties (22). It enhances antioxidant capacities by scavenging free radicals and increasing the activities of enzymes such as superoxide dismutase (SOD) and glutathione peroxidase (GPx), while inhibiting the NF-κB pro-inflammatory pathway (23). Recent studies have indicated that quercetin can enhance chemotherapy efficacy by regulating cellular signaling pathways (24–26), showing promise for cancer treatment. However, its application is limited due to its poor bioavailability (27). Notably, in hair regulation, quercetin influences follicle development through multiple pathways. It enhances DP cell viability by increasing NAD(P)H production and mitochondrial membrane potential, thereby optimizing cellular energy metabolism (28). Quercetin also prolongs anagen phase by upregulating the anti-apoptotic protein BCL2 and the proliferation gene *KI67* to regulate follicle cycle (29). In addition, it promotes the transcription and synthesis of growth factors such as bFGF, KGF, and VEGF, which are critical for maintaining follicle activity and periodic turnover. Mechanistically, quercetin activates the MAPK/CREB signaling pathway, inducing the phosphorylation of ERK, AKT, and CREB to regulate follicle-associated biological processes (30). Recent clinical studies have shown that quercetin stimulates the proliferation of keratinocytes in quiescent follicles and enhances dermal vascularization via HIF-1α activation, providing nutrients to follicles (31).

Quercetin exhibits broad biological activities and is compatible with modern livestock production. By prolonging anagen, protecting follicular cells from apoptosis, and enhancing vascularization, quercetin fulfills key requirements for sustaining SHFSC activity and promoting cashmere growth. As a naturally occurring flavonoid with a favorable safety profile, quercetin also shows promise for industrial use as a feed additive or topical agent to improve cashmere yield and fiber quality in a sustainable, residue-free manner. Therefore, investigating how quercetin regulates SHFSC proliferation and apoptosis may yield mechanistic insights with direct practical relevance for the cashmere industry.

Although quercetin's effects on follicle biology have been investigated, its role in regulating follicle development through SHFSCs in cashmere goats, particularly in relation to improving cashmere yield, remains poorly defined. To address this gap, we investigated the effects of quercetin on SHFSC proliferation and apoptosis in cashmere goats, with the aim of informing strategies to enhance cashmere production. Our findings highlight quercetin as a candidate compound for probing SHFSC-mediated regulation and underscore its potential for industrial application in cashmere goat production.

## 2 Materials and methods

### 2.1 Sample collection and cell culture

Three healthy 1-year-old cashmere goats were selected from the Yiwei White Goat Farm (Ordos, Inner Mongolia, China). In this study, all animals were reared under husbandry conditions following previous studies (32, 33), and food and water were available ad libitum. Skin biopsies (1 cm diameter) were collected from the dorsal region in September, when the hair follicle cycle had entered the anagen phase. SHFSCs were isolated following a published protocol (34). Briefly, under a stereomicroscope, secondary hair follicles were mechanically separated with forceps, the bulb region was dissected, and the tissue was digested overnight with collagenase type IV to facilitate attachment to culture dishes. Stem cells were further purified via sequential (gradient) digestion. Typically, follicle stem cells detach from the dish after ~5 min of trypsinization. SHFSC identity was verified using specific markers (CD34 and K19).

SHFSCs from Arbas cashmere goats were cultured in DMEM/F-12 (Biological Industries, Kibbutz Beit Haemek, Israel) supplemented with 4% fetal bovine serum, 14 ng/mL epidermal growth factor (PeproTech, Rocky Hill, NJ, USA), 0.4 ng/mL hydrocortisone (Monmouth Junction, NJ, USA), and 0.5 pg/mL ITS-X (Gibco BRL, Grand Island, NY, USA). Cells were maintained at 37 °C in a humidified 5% CO_2_ incubator, and the medium was replaced every other day. RNA and protein were extracted when cultures reached 70–80% confluence.

### 2.2 RNA extraction and quantitative real-time PCR detection

Passage-cultured SHFSCs were seeded and exposed to quercetin at the specified concentrations. After 48 h, total RNA was extracted using RNAiso Reagent (Takara Bio Inc., Shiga, Japan) in accordance with the manufacturer's instructions. RNA purity and concentration were assessed using a NanoDrop spectrophotometer (Thermo Fisher Scientific, Waltham, MA, USA). Complementary DNA was synthesized with the PrimeScript FAST RT Reagent Kit with gDNA Eraser (Takara Bio Inc.). Quantitative real-time PCR was performed with TB Green^Ⓡ^ Premix Ex Taq™ II (Takara Bio Inc.) on a CFX96 real-time PCR system (Bio-Rad Laboratories, Hercules, CA, USA). *GAPDH* served as the reference gene, and relative expression was calculated using the 2^−Δ*ΔCt*^ method. Primer sequences are provided in Supplementary material S1.

### 2.3 Protein extraction and western blot analysis

Passage-cultured SHFSCs were seeded and treated with quercetin at the specified concentrations for 48 h before lysis. Total protein was extracted using a mammalian protein extraction kit (CWBIO, Beijing, China) in accordance with the manufacturer's instructions. Protein concentration was determined using a bicinchoninic acid assay (Thermo Fisher Scientific). Equal amounts of protein (20 μg per lane) were separated through sodium dodecyl sulfate polyacrylamide gel electrophoresis and transferred to polyvinylidene difluoride membranes. Membranes were blocked in 5% (w/v) skimmed milk for 1 h at 37 °C and then incubated overnight at 4 °C with the primary antibodies (details in Supplementary material S1). After washing, membranes were incubated with horseradish peroxidase-conjugated secondary antibodies for 1 h at 37 °C. Chemiluminescent signals were recorded on a Tanon 5200 imaging system (Tanon, Shanghai, China). Band intensities were quantified in ImageJ (35) for statistical comparison between groups.

### 2.4 Flow cytometry analysis of the cell cycle and cell apoptosis

For cell-cycle analysis, passage-cultured SHFSCs were seeded in 6-well plates and treated with quercetin (specified concentrations) or vehicle for 48 h. The culture medium was collected, adherent cells were trypsinized for approximately 5 min, and the suspensions were pooled. Cells were pelleted at 1,500 × g for 5 min, washed once with ice-cold PBS, and fixed in ice-cold 70% ethanol at 4 °C for 12 h. After washing, cells were resuspended in propidium iodide (PI) staining buffer (Beyotime, Shanghai, China) containing RNase A, as per the manufacturer's instructions and incubated for 30 min at 37 °C in the dark. DNA content was analyzed on a FACSAria SORP flow cytometer (BD Biosciences, New Jersey, USA). Each group comprised three independent biological replicates (*n* = 3).

For apoptosis analysis, after 48 h of quercetin or vehicle treatment, cells were washed three times with 1 × PBS (pH 7.4), harvested via trypsinization, collected into 15-mL tubes, washed once with 1 × PBS, and resuspended in 1 × binding buffer (Solarbio, Beijing, China). Cells were stained with Annexin V–FITC (5 μL) and PI (10 μL) for 10 min at 37 °C in the dark and then analyzed on a FACSAria SORP flow cytometer. Acquisition settings were matched across groups, and data were analyzed using standard quadrant gating. Each group contained three independent biological replicates (*n* = 3).

### 2.5 Cell counting kit-8 (CCK-8) assay

SHFSCs were seeded into 96-well plates (100 μL medium per well) and treated with quercetin at the specified concentrations. Each treatment group comprised nine independent replicates. After 48 h of incubation at 37 °C in 5% CO_2_, 10 μL of CCK-8 reagent (Biosharp, Guangzhou, China) was added to each well and incubation continued for 4 h. Absorbance at 450 nm was then measured on a microplate reader (BioTek, Winooski, VT, USA), using medium-only wells for background subtraction.

### 2.6 EdU detection and immunofluorescence analysis

EdU labeling was performed following the manufacturer's instructions (EdU Cell Proliferation Kit, RiboBio, Guangzhou, China). Briefly, after the designated treatments, cells were incubated with serum-free medium containing 10 μM EdU for 24 h. Each group included three independent replicates. Fluorescence images were acquired on a Leica fluorescence microscope (Leica, Wetzlar, Germany), and EdU-positive rates were quantified in ImageJ.

For immunofluorescence, cells grown on coverslips in 24-well plates were fixed in 4% paraformaldehyde for 15 min, permeabilized in 0.5% Triton X-100 in PBS for 15 min, and blocked in PBS containing 0.5% Triton X-100 and 10% goat serum for 30 min at room temperature. After blocking, cells were incubated overnight at 4 °C with primary antibodies against CD34 and K19 (Proteintech, Wuhan, China). Dilution factors are provided in Supplementary material S2. The following day, cells were incubated with Cy5-conjugated secondary antibodies for 1 h at room temperature in the dark and then counterstained with DAPI for 5 min. Images were acquired using a confocal laser-scanning microscope(Nikon, Tokyo, Japan).

### 2.7 Transcriptome sequencing and bioinformatics analysis

For RNA-seq, passage-cultured SHFSCs were seeded and exposed to quercetin at the specified concentrations (or vehicle) for 48 h. They were then harvested for total RNA extraction and library preparation. Total RNA was extracted with TRIzol reagent in accordance with the manufacturer's instructions. RNA purity and quantity were assessed using a NanoDrop 2000 spectrophotometer (Thermo Fisher Scientific), and RNA integrity was evaluated on an Agilent 2100 Bioanalyzer (Agilent Technologies, Santa Clara, CA, USA). Strand-specific libraries were prepared with the VAHTS Universal V6 RNA-seq Library Prep Kit following the manufacturer's protocol. Sequencing and primary data processing were undertaken by Shanghai OE Biotech Co., Ltd. (Shanghai, China).

Libraries were sequenced on an Illumina NovaSeq 6000 platform to generate 150-bp paired-end reads. Raw FASTQ files were processed with fastp for adapter trimming and quality filtering, and clean reads were retained for downstream analyses. Reads were aligned to the reference genome using HISAT2 (2), followed by gene-level expression quantification. Principal component analysis was performed on count data to assess the consistency of biological replicates. Differential expression analysis was conducted with DESeq2, and genes with q-value < 0.05 or |log_2_ fold change| > 2 were designated as differentially expressed genes (DEGs). All statistical analyses were carried out in R (v4.5.0), and visualizations were generated with ggplot2. Functional enrichment was performed using the GO (http://geneontology.org/) and KEGG (http://www.kegg.jp/) online resources.

### 2.8 Statistical analysis

Data are presented as the mean ± standard error of the mean, with all results derived from three independent replicates. Comparisons between two groups were conducted using a two-tailed Student's *t*-test; unless otherwise specified, *P* < 0.05 was considered significant. All statistical analyses were performed using *t*-tests, and the significance was denoted as follows: ^*^P < 0.05, ^**^P < 0.01, ^***^P < 0.001, and “ns” indicates no significant difference.

## 3 Results

### 3.1 *In vitro* culture and identification of Arbas cashmere goat SHFSCs

**Figure 1:**
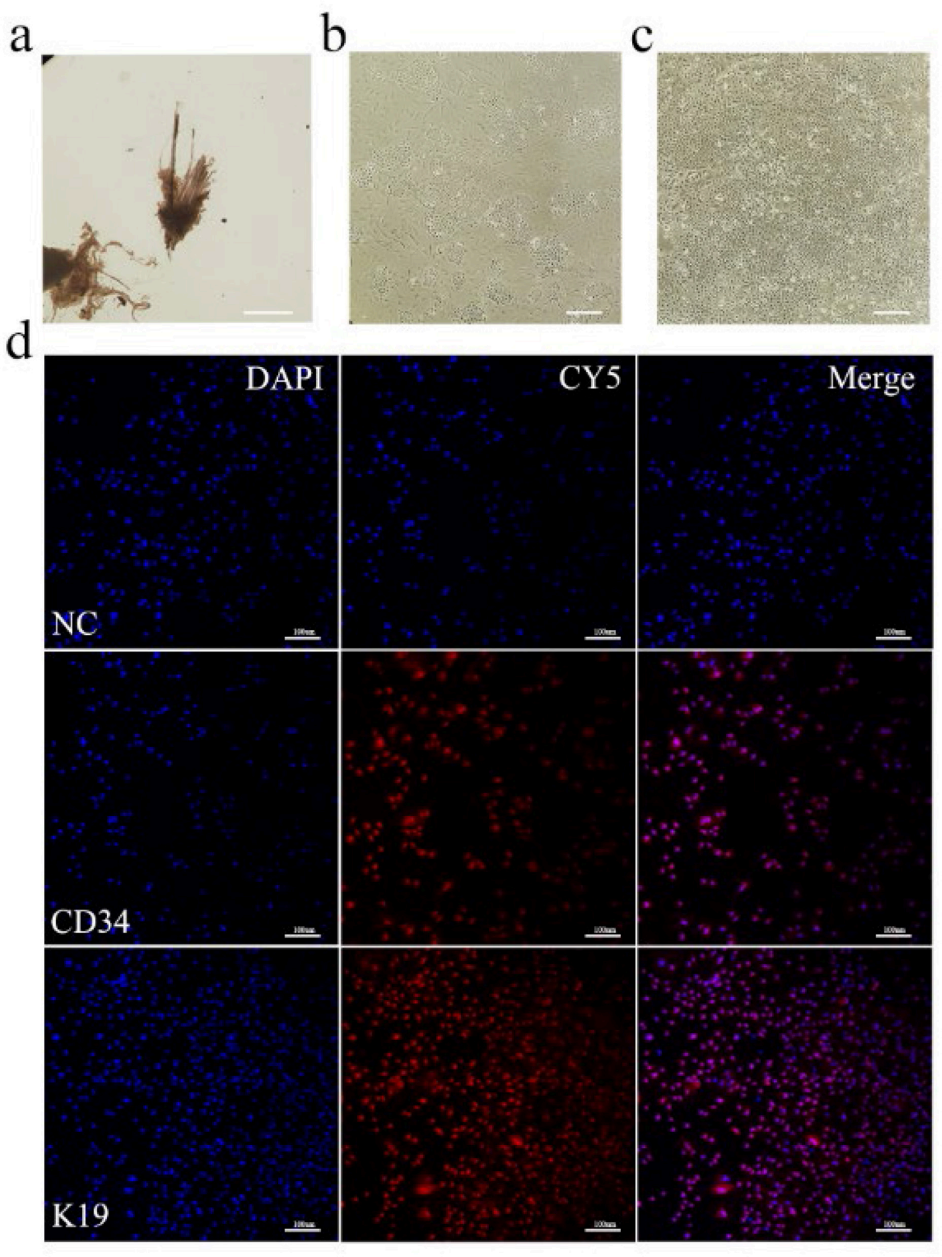
*In vitro* culture and identification of Arbas cashmere goat SHFSCs. **(a)** Primary and secondary follicles are isolated from skin tissue; **(b)** Adherent secondary follicle cells after culture; **(c)** Purified secondary follicle stem cells; **(d)** Immunofluorescence identification of SHFSCs using markers CD34 and K19. Nuclei were stained with DAPI, and secondary antibodies conjugated with the Cy5.5 fluorophore were used to detect the primary antibodies. The merged image shows the colocalization of the antibodies with the nuclei.NC refers to the negative control, that is, no primary antibody was incubated.

Dorsal skin samples were collected from Arbas cashmere goats during the anagen phase. Secondary follicles were isolated under a microscope (Figure 1a) and adherent cells were obtained after primary culture (Figure 1b). Primary and secondary follicle cells were digested with type IV collagen (36) to obtain purified SHFSCs (Figure 1c). To confirm their identity, immunofluorescence staining for the SHFSC surface markers CD34 and K19 was performed, with positive signals shown in red (Figure 1d). The purified SHFSCs exhibited a cobblestone-like morphology, small size, rapid division, and high viability.

### 3.2 Quercetin promotes the proliferation of Arbas cashmere goat SHFSCs

**Figure 2:**
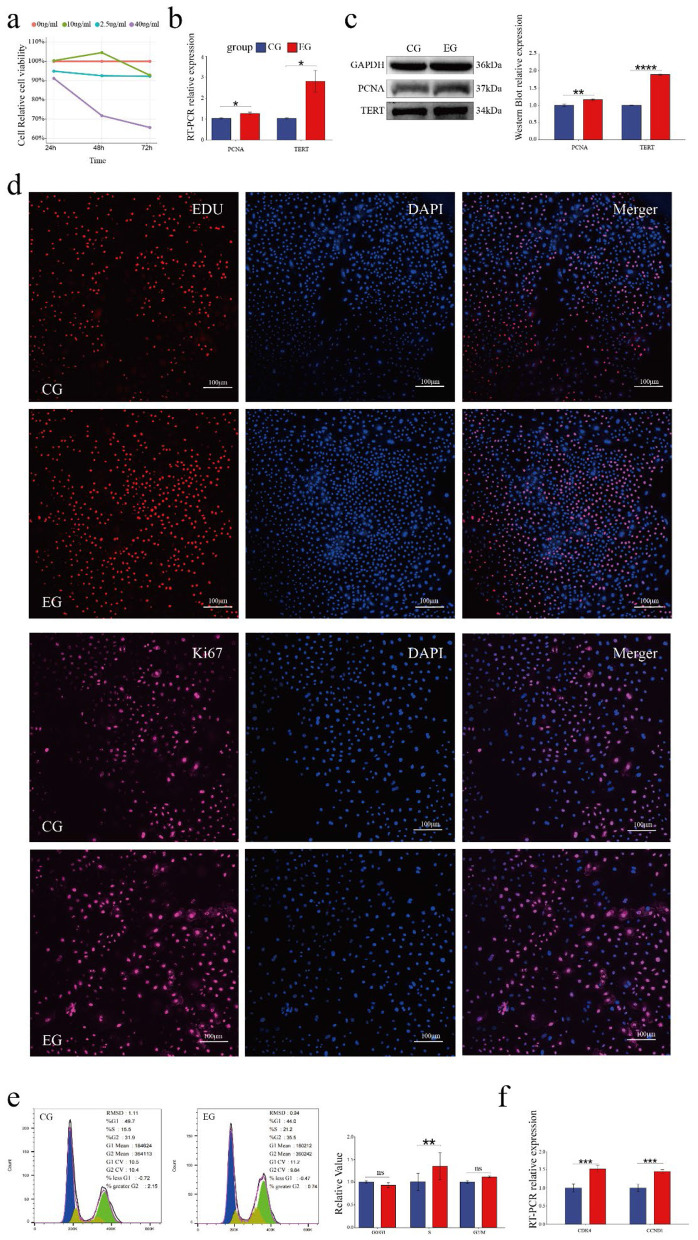
Quercetin promotes the proliferation of Arbas cashmere goat SHFSCs. **(a)** CCK-8 assay showing the proliferation activity of follicle stem cells following treatment with different quercetin concentrations and durations; **(b)** RT-qPCR detection of *PCNA* and *TERT* mRNA expression in the control group (CG) and experimental group (EG); **(c)** Western blot analysis of PCNA and TERT protein expression in the CG and EG, with quantitative analyses via grayscale scanning. GAPDH served as the internal control; **(d)** EdU assay and KI67 immunofluorescence under a fluorescence microscope, with quantitative analyses performed using fluorescence intensity; **(e)** Flow cytometry analysis of cell cycle phases in the CG and EG, with quantitative comparisons of cell percentages; **(f)** RT-qPCR detection of *DK4* and *CCND1* mRNA expression in the CG and EG.

Quercetin treatment at specific concentrations and durations promoted follicle stem cell proliferation. To determine the optimal conditions, different concentrations of quercetin (0, 2.5, 10, and 40 μg/mL) were applied for varying durations (24 h, 48 h, and 72 h). The highest cell viability was observed with 10 μg/mL quercetin for 48 h (Figure 2a). Therefore, this condition was selected as the experimental group (EG), while 0 μg/mL quercetin for 48 h was used as the control group (CG).

RT-qPCR analyses showed significantly increased mRNA expression levels of the proliferation markers, proliferating cell nuclear antigen (PCNA) and telomerase reverse transcriptase (TERT), in the EG compared to that in the CG (Figure 2b). This observation was consistent with the elevated protein levels of PCNA and TERT (Figure 2c). EdU assays revealed significantly more EdU-positive cells in the EG than in CG and confocal microscopy showed the upregulated expression of the proliferation marker KI67, further confirming enhanced proliferation (Figure 2d). Cell cycle analyses indicated a significant increase in the number of S-phase cells in the EG (from 15.5% to 21.2%; Figure 2e). RT-qPCR analyses also showed the upregulated expression of the cell cycle-related genes *CDK4* and *CCND1* in the EG (Figure 2f). Collectively, these results demonstrate that quercetin promotes the proliferation of goat skin follicle stem cells.

### 3.3 Quercetin inhibits the apoptosis and modulates the physiological status of Arbas cashmere goat SHFSCs

**Figure 3:**
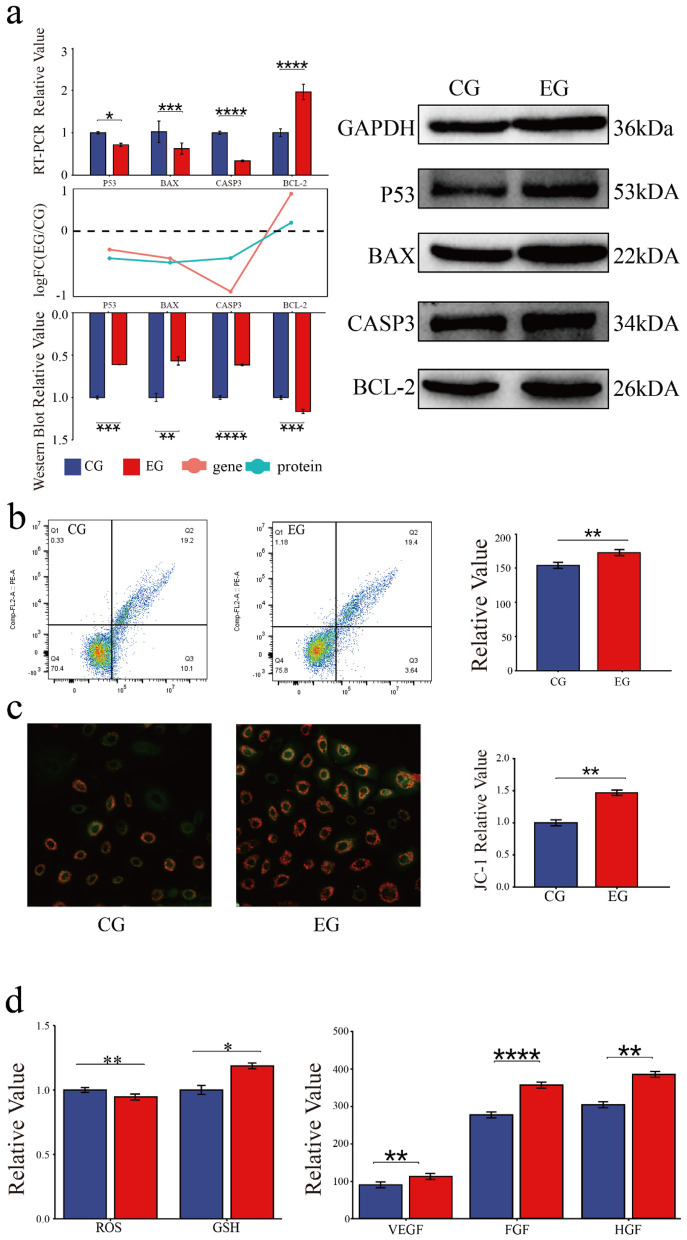
Quercetin inhibits the apoptosis and modulates the physiological status of Arbas cashmere goat SHFSCs. **(a)** RT-qPCR and western blot analyses of p53, BAX, *CASP3*, and BCL2 expression in the CG and EG, with quantitative bar graphs and trend line graphs. GAPDH served as the internal control; **(b)** Flow cytometry analysis of apoptosis in the CG and EG using Annexin V-FITC/PI staining, with quantitative analyses of late apoptotic cells (Q3); **(c)** Fluorescence intensity of JC-1 staining under a microscope; **(d)** Quantification of glutathione (GSH), reactive oxygen species (ROS), VEGF, FGF, and HGF levels in the CG and EG using a microplate reader, with quantitative bar graphs.

The role of quercetin in the apoptosis of goat secondary skin follicle stem cells was further investigated. The mRNA levels of the pro-apoptotic genes *BAX, TP53*, and *CASP3* were downregulated in quercetin-treated cells, whereas the expression of the anti-apoptotic gene *BCL2* was upregulated. Consistently, the protein levels of BAX, p53, and *CASP3* were downregulated and that of BCL2 was upregulated in the EG. The RNA and protein expression trends are shown as bar and line graphs in Figure 3a. Annexin V-FITC/PI staining further showed that quercetin inhibited apoptosis and significantly reduced the proportion of late apoptotic cells (Figure 3b), confirming its anti-apoptotic effects.

The effects of quercetin on physiological status of SHFSCs were also assessed. JC-1 staining showed increased mitochondrial membrane potential in the EG compared to that in the CG (Figure 3c), indicating improved energy metabolism. Additionally, quercetin reduced reactive oxygen species (ROS) levels, increased the glutathione (GSH) content, and promoted the secretion of the growth factors VEGF, FGF, and HGF (Figure 3d). These results demonstrate that quercetin enhances antioxidant capacity, sustains energy metabolism, and promotes growth factor secretion, thereby supporting SHFSC proliferation.

### 3.4 Quercetin alters the transcriptome of Arbas cashmere goat SHFSCs

**Figure 4:**
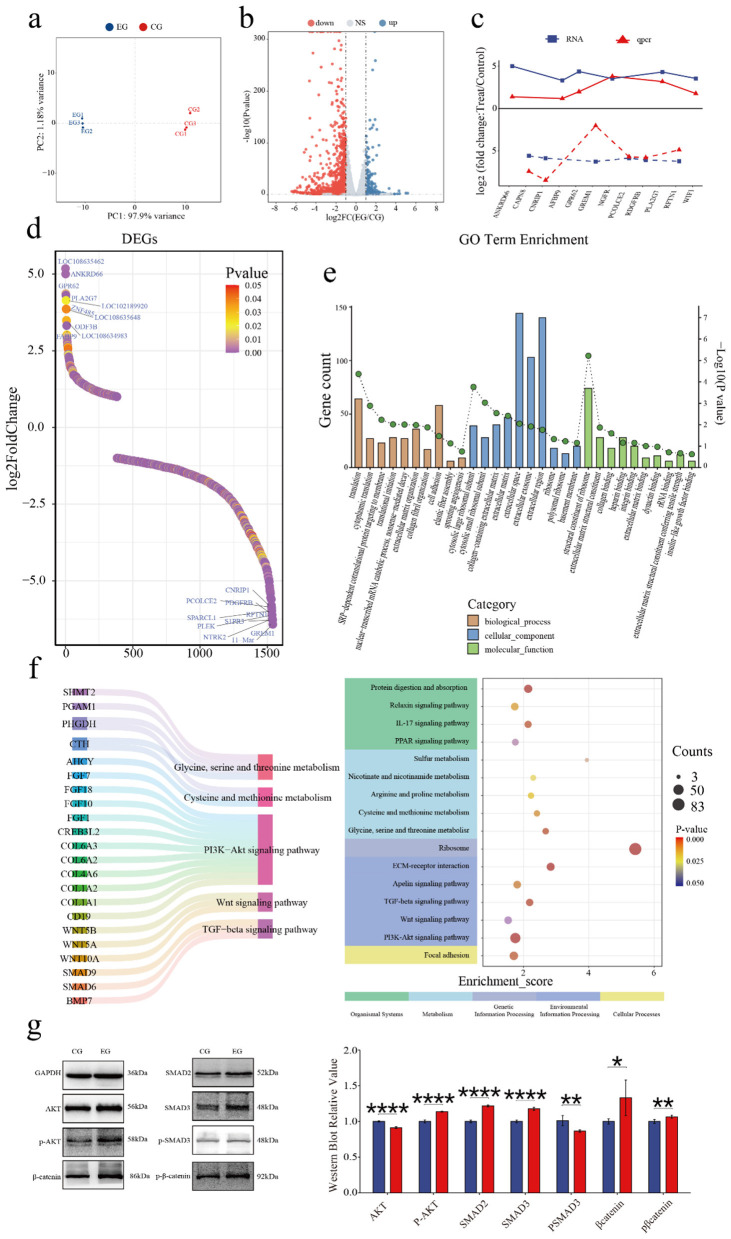
Quercetin alters the transcriptome of Arbas cashmere goat SHFSCs. **(a)** Principal component analysis (PCA) plot of RNA-seq data from the CG and EG; **(b)** Volcano plot of the differentially expressed genes (DEGs) between the CG and EG; **(c)** Trend comparisons of the gene expression between the RNA-seq and qPCR data; **(d)** Sorted logFC values of the DEGs; **(e)** Gene Ontology (GO) term enrichment analysis of the DEGs; **(f)** Kyoto Encyclopedia of Genes and Genomes (KEGG) bubble plot and Sankey diagram showing pathway–gene relationships; **(g)** Western blot analysis of the key pathway proteins in the CG and EG, with quantitative bar graphs created via grayscale scanning.

To investigate quercetin's effects on the SHFSC transcriptome, high-throughput RNA sequencing was performed on SHFSCs from EG and CG. After quality control and sequence alignment, 16,751 expressed genes were identified. A principal component analysis (PCA) distinguished gene expression profiles between the EG and CG, showing high intragroup reproducibility and confirming experimental consistency and reliability (Figure 4a).

The DEGs were filtered using the following criteria: |logFC|≥1 and *P* < 0.05, identifying 1,540 DEGs in the EG vs. CG, including 1,157 upregulated and 383 downregulated genes (Figure 4b). To validate the accuracy of the RNA-seq, six upregulated and six downregulated genes were randomly selected for qPCR verification. The logFC trends between the RNA-seq and qPCR results were consistent and visualized in a trend line comparison graph (Figure 4c). The top 10 upregulated and downregulated DEGs were identified as potential key genes, with logFC values shown in a sorted graph (Figure 4d). Notably, *ANKRD66* and *PLA2G7* were significantly upregulated. ANKRD66 is associated with enhanced cell proliferation in tumor cells (37), whereas PLA2G7 catalyzes phospholipid hydrolysis to produce arachidonic acid, participating in inflammatory signaling and lipid metabolism (38, 39). In contrast, *GREM1* is found to be downregulated. GREM1 is a BMP antagonist that inhibits BMP signaling, which typically suppresses proliferation and promotes differentiation (40). Its reduced expression may enhance BMP signaling and inhibit proliferation, however, this effect may have been offset by the pro-proliferative effects of quercetin.

A GO enrichment analysis explored the biological functions associated with the DEGs, including biological processes (BPs), cellular components (CCs), and molecular functions (MFs; Figure 4e). In BPs, “cytoplasmic translation” and “translational initiation” were enriched, indicating that quercetin promotes protein synthesis to support proliferation. In MFs, “insulin-like growth factor binding” was enriched. Insulin-like growth factors, such as IGF-1 and IGF-2, regulate proliferation via the PI3K–Akt and RAS–MAPK pathways, consistent with the observed pro-proliferative effects.

A KEGG enrichment analysis (Figure 4f) showed that the DEGs were enriched in various pathways including Cellular Processes, Environmental Information Processing, Metabolism, Genetic Information Processing, and Organismal Systems. Key pathways included PI3K–Akt, Wnt, and TGF-β signaling, which are part of Environmental Information Processing. Additionally, under Metabolism, pathways related to cysteine/methionine metabolism and glycine/serine/threonine metabolism were enriched. These pathways are critical for follicle development and SHFSC proliferation. Key regulatory genes identified include the COL6A family and CD19 in the PI3K–Akt signaling pathway (41, 42); Wnt family genes in Wnt signaling; and SMAD family genes and BMP7 in TGF-β signaling. Additionally, CTH and AHCY reduced ROS levels and enhanced the antioxidant capacity through the cysteine/methionine metabolism pathway (43, 44). Western blot analyses of the key pathway proteins (Figure 4g) confirmed differential expression levels consistent with the sequencing data, supporting quercetin's role in activating the PI3K–Akt, Wnt, and TGF-β signaling pathways.

In summary, the transcriptome analysis revealed that quercetin promotes SHFSC proliferation and metabolism via multiple pathways, including PI3K–Akt, Wnt, TGF-β, and PSAT1-related pathways, clarifying its molecular mechanisms.

## 4 Discussion

This study found that quercetin promotes SHFSC proliferation in a concentration- and time-dependent manner, with optimal effects at treatment with 10 μg/mL quercetin for 48 h. This is consistent with previous findings in human DP cells (34). Enhanced proliferation was revealed by an increased number of S-phase cells and upregulated PCNA and TERT expressions, suggesting that quercetin accelerates DNA replication and maintains telomere stability. Notably, upregulated Cyclin D1 and CDK4 expression—key regulators of the G1/S transition—may relieve the Rb-mediated inhibition of E2F transcription factors, activating DNA replication-related genes (45, 46).

Transcriptome analysis and western blot showed that quercetin activates the PI3K–Akt and Wnt/β-catenin pathways. PI3K–Akt activation may inhibit β-catenin degradation via GSK3β phosphorylation, promoting nuclear translocation and the transcription of proliferation genes (47). This is supported by observed increase in β-catenin and phosphorylation levels. Wnt10b, a key anagen inducer, may synergize with PI3K–Akt to drive the SHFSC transition from quiescence to proliferation (48).

Quercetin may modulate SHFSC proliferation via additional signaling axes, including TGF-β, MAPK, and AMPK. These pathways govern cell-cycle progression, metabolic adaptation, and oxidative-stress responses. In particular, TGF-β signaling may cooperate with PI3K/Akt to shape stem-cell behavior, promoting proliferation while constraining apoptosis. Moreover, quercetin's effects on mitochondrial dynamics, evidenced by increased mitochondrial membrane potential and reduced ROS, suggest support of cellular energy homeostasis and enhanced mitochondrial function during proliferative activity.

Quercetin downregulated the expression of pro-apoptotic genes (*BAX, TP53*, and *CASP3*) and upregulated that of *BCL2*, consistent with its anti-apoptotic effects in tumor cells (49, 50). Mechanistically, JC-1 staining revealed increased mitochondrial membrane potential, reduced cytochrome c release, and caspase cascade activation. As a natural antioxidant, quercetin reduces ROS levels and increases GSH levels, mitigating oxidative damage to DNA and mitochondria (51). An enhanced antioxidant capacity may also promote the secretion of VEGF and FGF, which further stimulates SHFSC proliferation via paracrine effects.

Quercetin's antioxidant and anti-inflammatory properties may also enhance hair-follicle vascularization. By reducing oxidative stress, it not only protects SHFSCs but also supports overall follicle health, which could be critical for promoting cashmere production in goats. In addition, quercetin's modulation of mitochondrial function and cellular energy metabolism may improve the functional fitness of SHFSCs, thereby sustaining cell proliferation throughout the hair-growth cycle.

Transcriptome analyses identified 1,540 DEGs, with *ANKRD66* and *PLA2G7* being significantly upregulated. ANKRD66, an ankyrin repeat family member, may regulate cell proliferation via cytoskeletal dynamics, consistent with its proliferative role in cancer (52). PLA2G7 generates arachidonic acid, a precursor of proinflammatory and proliferative prostaglandins, aligned with an improved inflammatory microenvironment (22). Downregulated GREM1 expression may enhance BMP signaling (typically pro-differentiation/anti-proliferation); however, this effect may be offset by quercetin-activated pathways (11).

KEGG enrichment analyses highlighted the PI3K–Akt, Wnt, and TGF-β pathways. The reduced phosphorylation of SMAD2/3 in TGF-β signaling may weaken the transcriptional inhibition of Cyclin D1, thereby accelerating the cell cycle (53–55). The activation of cysteine/methionine metabolism may provide methyl donors to support DNA/histone methylation, whereas CTH and AHCY reduce ROS, enhance the antioxidant capacity, and regulate proliferation-related genes (56, 57)

These findings elucidate the multifaceted mechanisms by which quercetin regulate SHFSC proliferation. By modulating key signaling pathways, including PI3K/Akt, Wnt/β-catenin and TGF-β, quercetin emerges as a promising natural modulator of hair-follicle stem-cell activity. In parallel, its enhancement of mitochondrial function, attenuation of oxidative stress and stimulation of growth-factor secretion further support its potential as a therapeutic intervention to improve cashmere yield and fiber quality.

## 5 Conclusions

Our results demonstrate that quercetin plays a critical role in regulating follicle stem cell proliferation and apoptosis, specifically promoting proliferation and inhibiting apoptosis. It exerts these effects via multiple signaling pathways, including PI3K-Akt, Wnt, and TGF-β-related pathways, enhancing SHFSCs proliferation and metabolism. These findings provide valuable insights into developing a regulatory strategy for improving cashmere production from goats.

## Data Availability

The data presented in the study are deposited in the NCBI repository, accession number PRJNA1346451.
