# Supplementary material for: Quercetin promotes production of secondary hair follicle stem cells in cashmere goat: a mechanistic study
Source: Front Vet Sci. 2025 Oct 31;12:1689059. doi: 10.3389/fvets.2025.1689059 (PMC12616863; doi:10.3389/fvets.2025.1689059)
Supplement: Supplementary file 1 [file Data_Sheet_1.zip › supplement/Supplementary S1.docx]

Table S1 Primer Sequences

| Gene name | Forward primers 5’—3’ | Revers primers 5’—3’ |
| --- | --- | --- |
| *GAPDH* | GGTCGGAGTGAACGGAT | TCTGCCTTGACTGTGCC |
| *PCNA* | CTTGAAGAAAGTGCTGGAG | TGGACATGCTGGTGAGG |
| *TERT* | CAGCCCCTCCGCTCTACTTC | GCGCACGCAGTACGTATTCT |
| *P53* | TCAGGAGACATTTTCCGACT | GAGGCTCTGGCATTTGG |
| *BAX* | TCCGACGGCAACTTCAA | ACAGGGACAGCAGGCAC |
| *BCL-2* | CCTGTGGATGACCGAGTA | GACAGCCAGGAGAAATCA |
| *CDK4* | GCATGTGGACCAAGACCTCA | ACTGGCGCATCAGATCCTTT |
| *Cyclin D1* | ATCAGATGTGACCCGGACTG | CCCTCAAATGTTCACGTCGC |
| *CASPASE3* | GGAGCAGTTTTATTTGCGTGC | TTCTGCAACAGTCCCCTCTG |
| *PLA2G7* | AAAGCCTTGTCCCGGTTCC | TACTGACCCATGCTGGGGAT |
| *GPR62* | TCAAATTCCGGCAGTAGACAAGA | TGGCTCTCTCTTCCACACCA |
| *ANKRD66* | CCTCTCAGTTCCCGCAAAGA | CAGGCTGTTTGGAACTCCCC |
| *NGFR* | TTGCCTTCAAGAGGTGGAACA | CACTGTGCAGCTTTTCTCCC |
| *GREM1* | CGCGTTGACAGCATGAGC | TCTGCTCGGAGTCATTGTGC |
| *PDGFRB* | CCATTCTCCTGAGGCTCTGC | CGCATGGTATCCTTGCTGC |
| *CMAH* | ATTTCTGCCTTGGGGGAGTG | CTGTTTGTTCGATGCTGCCC |
| *WIF1* | CCTGTCAACAAGCTGAGTGC | AACACATAGCCCGCCATTCA |
| *S1PR3* | CCTTCTCGGATAGAGGAGCC | AGGTATAAGGTCTGGGGCGT |
| *PCOLCE2* | CGTGGCTGTGTTTAACGGTG | AGATACAATTGGCGCAGGGG |
| *FABP9* | TGAAAGAGCTGGGAGTGAGT | TCCCCGTCGGCACTAATAAT |
| *RFTN1* | TTGGCTTGGTCCACGATTCC | ACAATAGCGTCGTAGCCCTG |
| *CNRIP1* | CAGATCACCATGCCGTTCAC | TCGTTGGGCTTGCATTCGTA |
| *CAPN8* | CGTGAGGTACTTGGGCCAG | TGGGGCACAATTCCGTGG |
| *PLA2G7* | AAAGCCTTGTCCCGGTTCC | TACTGACCCATGCTGGGGAT |
