# Supplementary material for: Quercetin promotes production of secondary hair follicle stem cells in cashmere goat: a mechanistic study
Source: Front Vet Sci. 2025 Oct 31;12:1689059. doi: 10.3389/fvets.2025.1689059 (PMC12616863; doi:10.3389/fvets.2025.1689059)
Supplement: Supplementary file 1 [file Data_Sheet_1.zip › supplement/Supplementary S2.docx]

Table S2 Antibody Information

| Antibody Name | Company | Catalog Number | Recommended dilution ratio |
| --- | --- | --- | --- |
| Smad2 Polyclonal antibody | Proteintech | 12570-1-AP | 1:2000 |
| Smad3 Polyclonal Antibody | Proteintech | 30130-1-AP | 1:1000 |
| Smad3(phospho Ser204)Polyclonal Antibody | Immunonway | YP0363 | 1:500 |
| AKT Polyclonal antibody | Proteintech | 10176-2-AP | 1:2000 |
| Phospho-AKT (Ser473) Polyclonal antibody | Proteintech | 28731-1-AP | 1:1000 |
| β-Catenin Polyclonal antibody | Proteintech | 51067-2-AP | 1:5000 |
| Phospho-β-Catenin | Cell signaling | 9561T | 1:1000 |
| PCNA Polyclonal antibody | Proteintech | 10205-2-AP | 1:1000 |
| Caspase 3 Polyclonal antibody | Proteintech | 19677-1-AP | 1:500 |
| P53 Polyclonal antibody | Proteintech | 10442-1-AP | 1:5000 |
| HRP-conjugated Goat Anti-Rabbit IgG(H+L) | Proteintech | SA00001-2 | 1:2000 |
| TERT Antibody | Abmart | TD7129M | 1:500 |
| Bax Rabbit pAb | Immunoway | YT0455 | 1:500 |
| Bcl-2 Rabbit pAb | Immunoway | YT0470 | 1:500 |
| CD34 Polyclonal antibody | Proteintech | 14486-1-AP | 1:1000 |
| Cytokeratin 19 Polyclonal antibody | Proteintech | 10712-1-AP | 1:10000 |
| DAPI solution | Proteintech | PR30021 | 1:1000 |
