# Supplementary material for: Quercetin promotes production of secondary hair follicle stem cells in cashmere goat: a mechanistic study
Source: Front Vet Sci. 2025 Oct 31;12:1689059. doi: 10.3389/fvets.2025.1689059 (PMC12616863; doi:10.3389/fvets.2025.1689059)
Supplement: Supplementary file 2 [file Data_Sheet_2.zip › WB/Explanatory notes about the original picture.pdf]

In the original experiment, we used the concentration of 0, 10, and 40 μg/mL of quercetin, but we found that the concentration of 40 μg/mL was not ideal, so we abandoned this concentration, and the three lanes corresponded to 0, 10, and 40 μg/mL respectively, and only the first two lanes were applied in our manuscript.

Western blot in manuscript figure 2.c

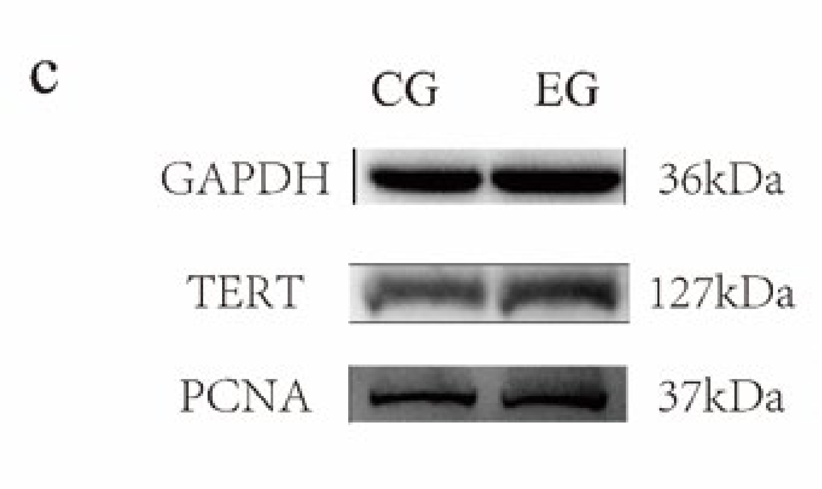

**Protein name**	**Raw plot**	**Filename**
GAPDH		GAPDH.tif
TERT	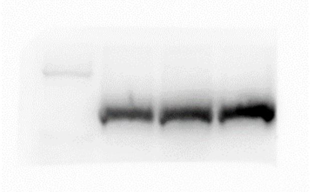	TERT.tif
PCNA	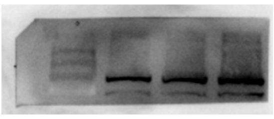	PCNA .tif

Western blot in manuscript figure 3.a

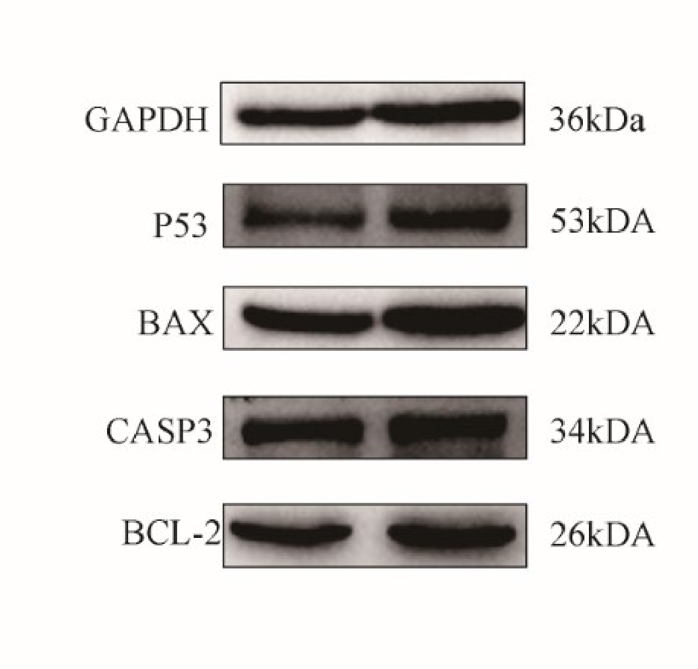

Protein name	Raw plot	Filename
GAPDH	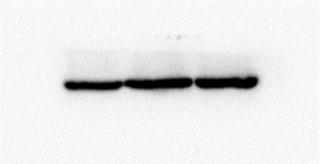	GAPDH2.tif
P53	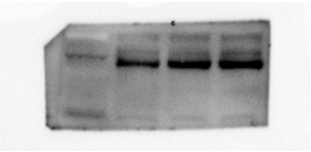	P53.tif
BAX	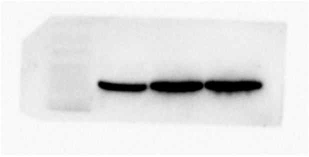	BAX.tif
CASP3	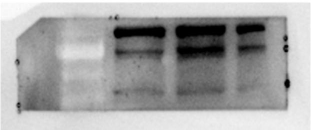	CASP33.Tif
BCL2	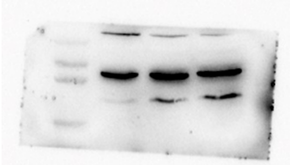	BCL-2.tif

Western blot in manuscript figure 4.g

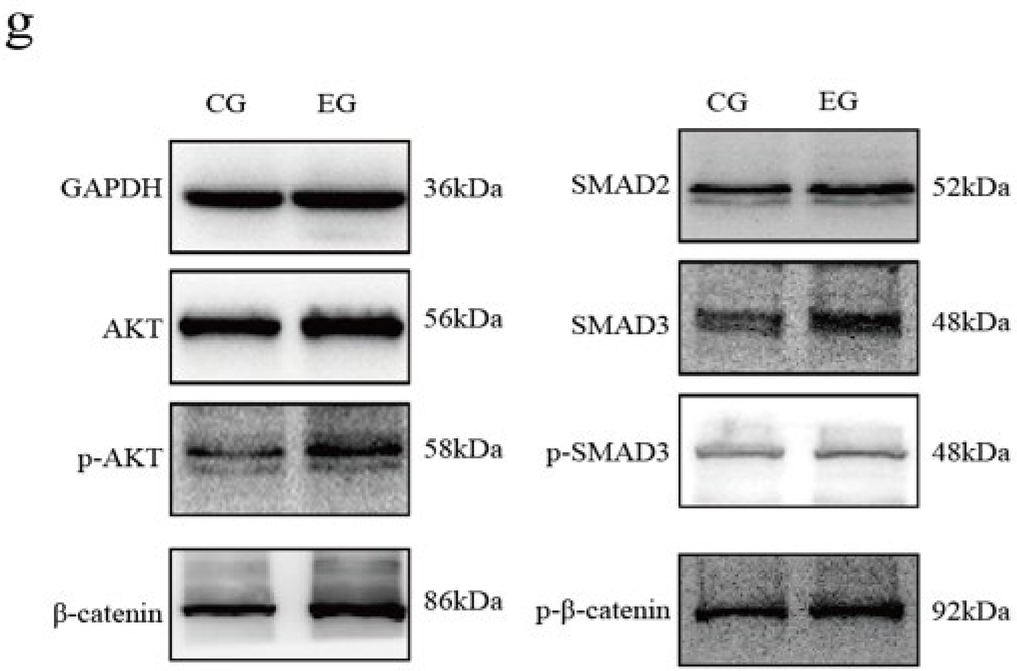

Protein name	Raw plot	Filename
GAPDH		GAPDH3.tif
AKT	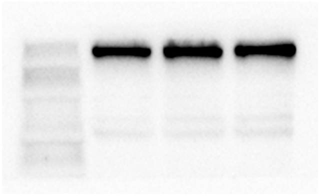	AKT.tif
P-AKT	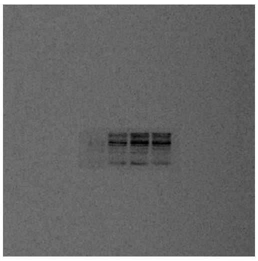	P-AKT.tif

SMAD2	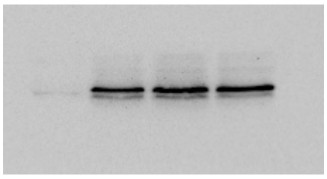	SMAD2.tif
SMAD3	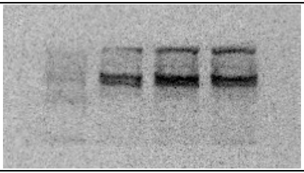	SMAD3.tif
β-catenin	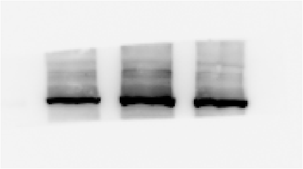	β-catenin.tif
P-β-catenin	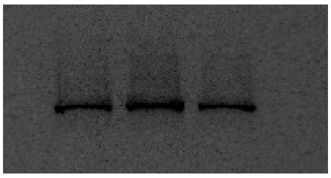	P-β-catenin.tif
