# Supplementary figures and images for: Quercetin promotes production of secondary hair follicle stem cells in cashmere goat: a mechanistic study
Source: Front Vet Sci. 2025 Oct 31;12:1689059. doi: 10.3389/fvets.2025.1689059 (PMC12616863; doi:10.3389/fvets.2025.1689059)

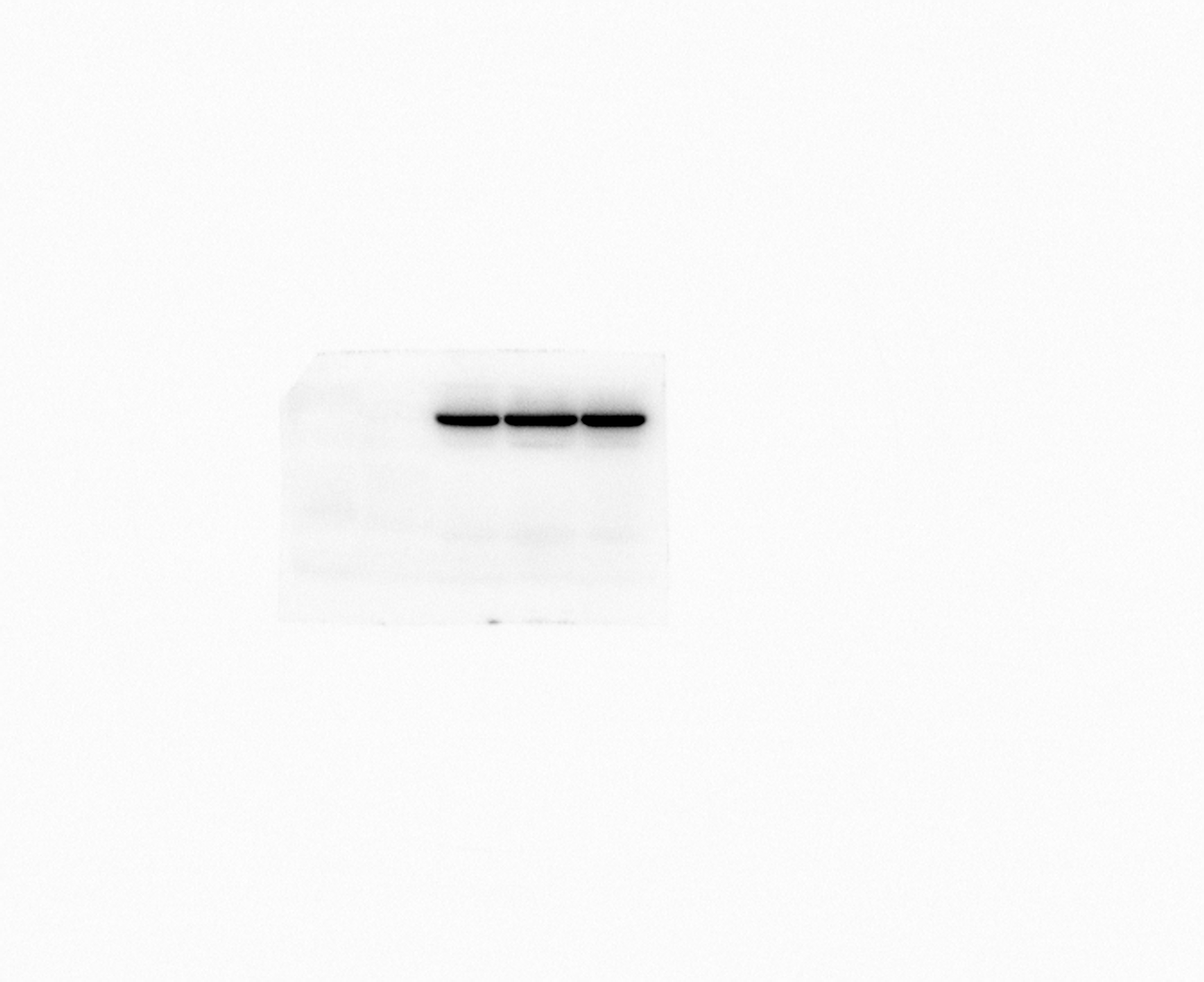

Supplement: Supplementary file 2 [file Data_Sheet_2.zip › WB/figure_2wb/GAPDH.tif]

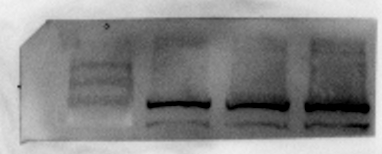

Supplement: Supplementary file 2 [file Data_Sheet_2.zip › WB/figure_2wb/PCNA .tif]

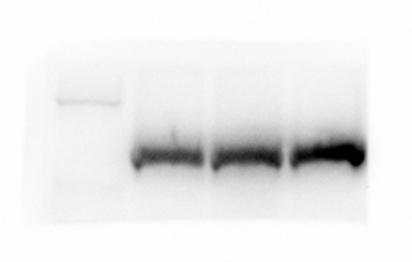

Supplement: Supplementary file 2 [file Data_Sheet_2.zip › WB/figure_2wb/TERT.tif]

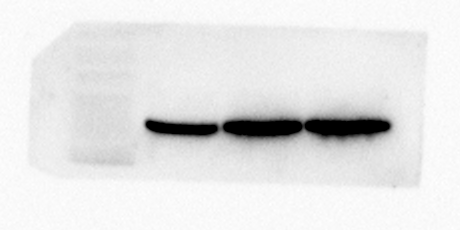

Supplement: Supplementary file 2 [file Data_Sheet_2.zip › WB/figure_3wb/BAX.tif]

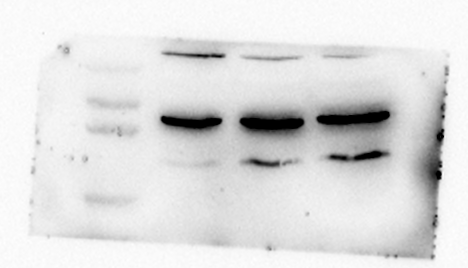

Supplement: Supplementary file 2 [file Data_Sheet_2.zip › WB/figure_3wb/BCL-2.tif]

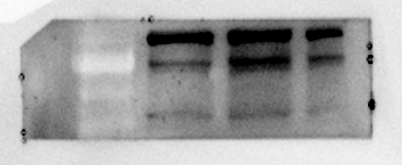

Supplement: Supplementary file 2 [file Data_Sheet_2.zip › WB/figure_3wb/CASP33.Tif]

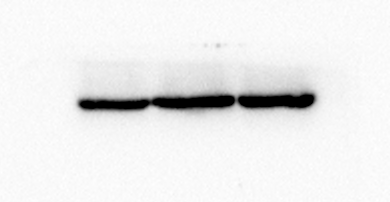

Supplement: Supplementary file 2 [file Data_Sheet_2.zip › WB/figure_3wb/GAPDH2.tif]

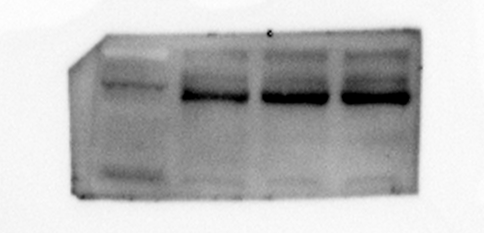

Supplement: Supplementary file 2 [file Data_Sheet_2.zip › WB/figure_3wb/P53.tif]

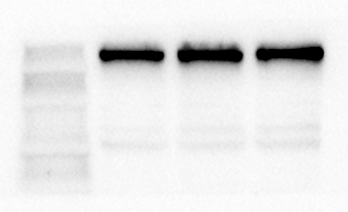

Supplement: Supplementary file 2 [file Data_Sheet_2.zip › WB/figure_4wb/AKT.tif]

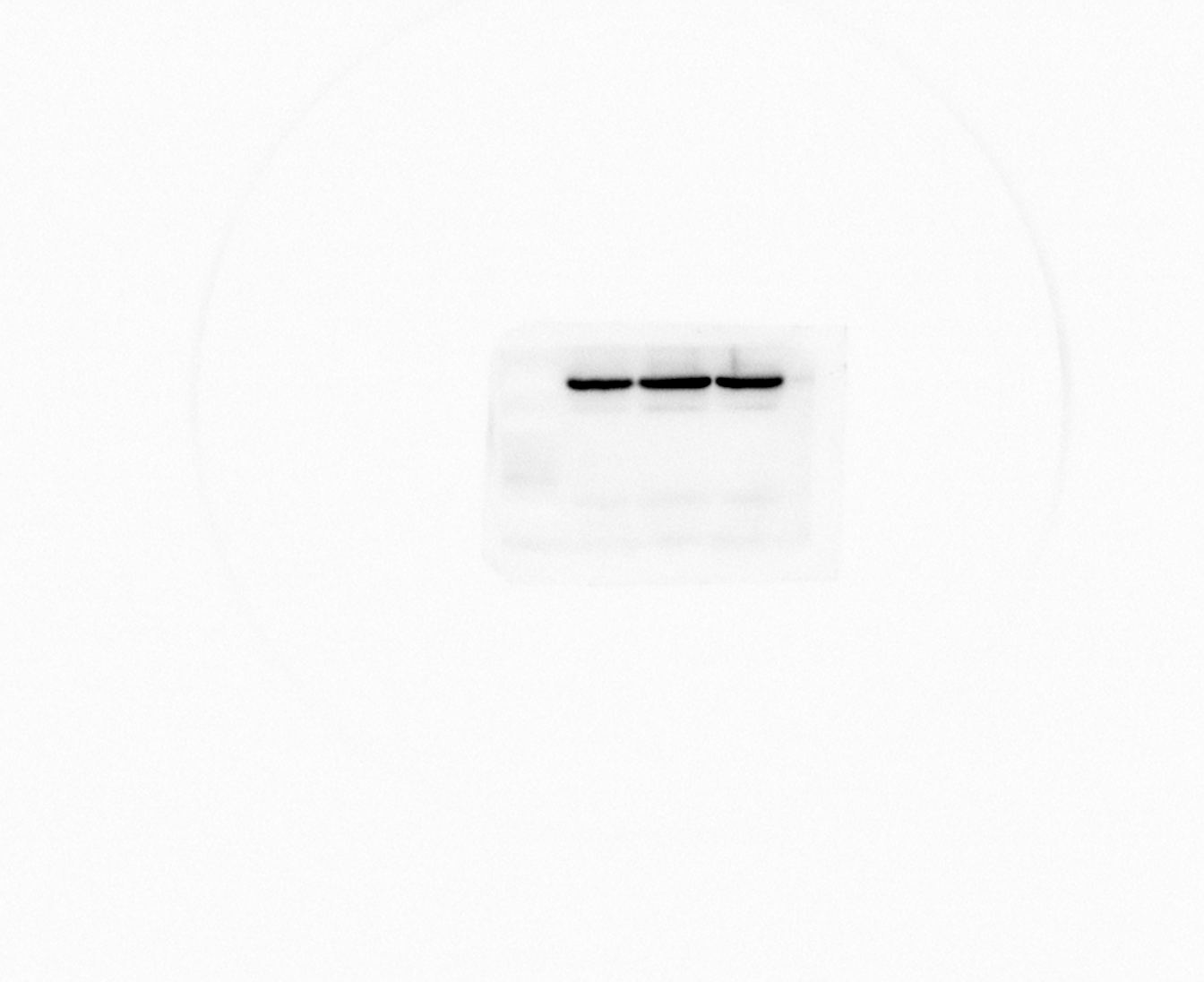

Supplement: Supplementary file 2 [file Data_Sheet_2.zip › WB/figure_4wb/GAPDH3.tif]

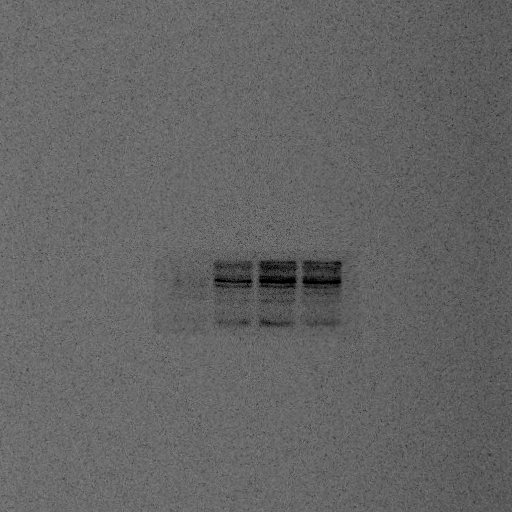

Supplement: Supplementary file 2 [file Data_Sheet_2.zip › WB/figure_4wb/P-AKT.tif]

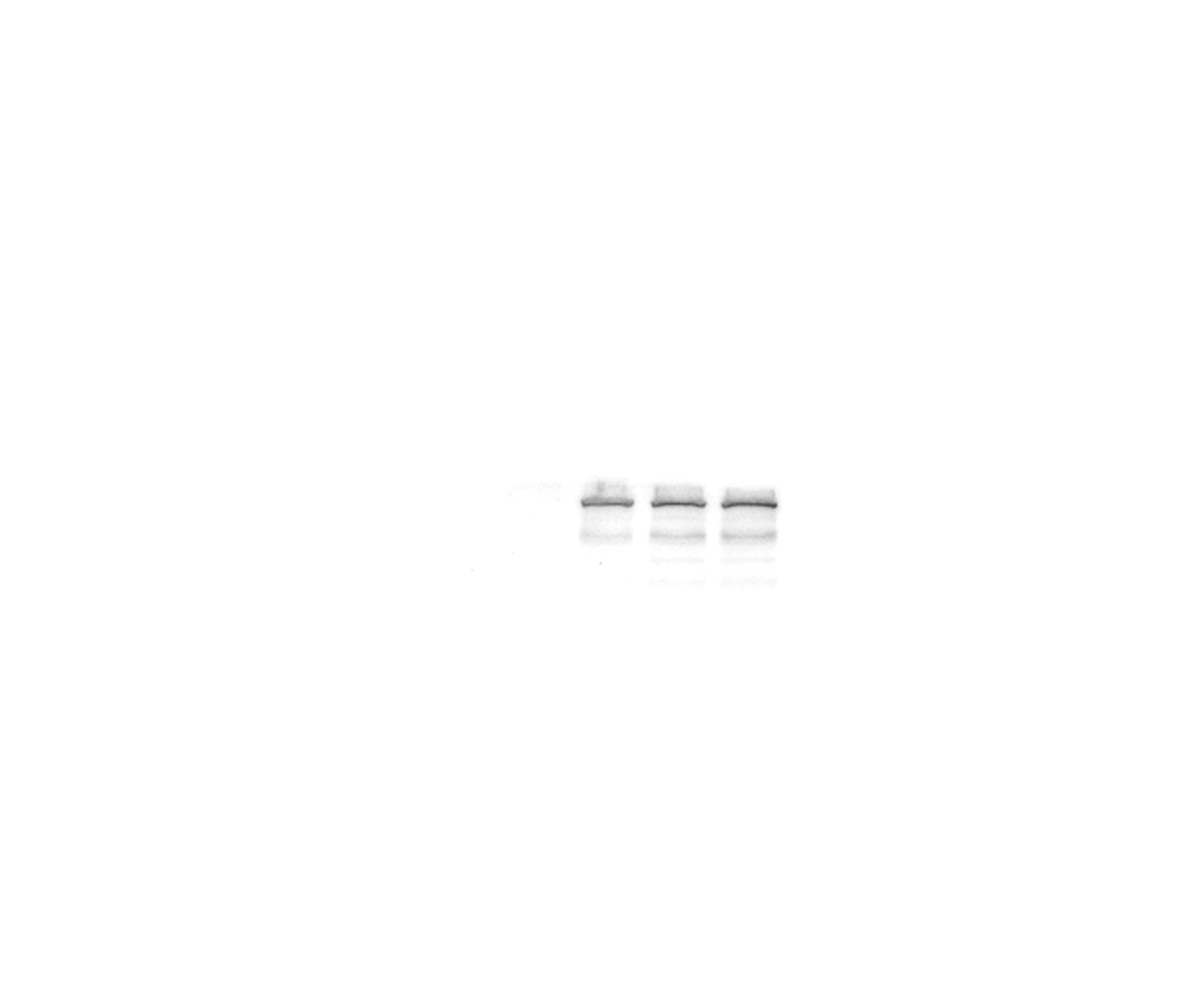

Supplement: Supplementary file 2 [file Data_Sheet_2.zip › WB/figure_4wb/P-SMAD3.Tif]

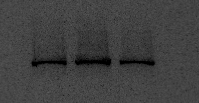

Supplement: Supplementary file 2 [file Data_Sheet_2.zip › WB/figure_4wb/P-β-catenin.tif]

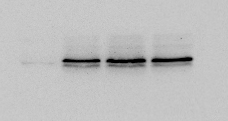

Supplement: Supplementary file 2 [file Data_Sheet_2.zip › WB/figure_4wb/SMAD2.tif]

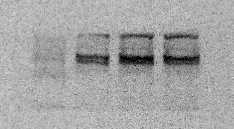

Supplement: Supplementary file 2 [file Data_Sheet_2.zip › WB/figure_4wb/SMAD3.tif]

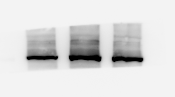

Supplement: Supplementary file 2 [file Data_Sheet_2.zip › WB/figure_4wb/β-catenin.tif]
